# Epidemiology and Early Bacteriology of Extremely Severe Burns from an LPG Tanker Explosion in Eastern China

**DOI:** 10.1007/s44197-022-00066-0

**Published:** 2022-09-27

**Authors:** Ronghua Jin, Min Yang, Tingting Weng, Jiaming Shao, Sizhan Xia, Chunmao Han, Xingang Wang

**Affiliations:** 1 Department of Burns and Wound Care Center, The Second Affiliated Hospital of Zhejiang University College of Medicine, Hangzhou, China; 2 Key Laboratory of the Diagnosis and Treatment of Severe Trauma and Burn of Zhejiang Province, Hangzhou, China

**Keywords:** Bacteriology, Epidemiology, Burns, Liquefied petroleum gas, Fungi

## Abstract

The incidence of liquefied petroleum gas (LPG)-related accidents in China has increased over the recent years. In addition, infection remains a big challenge in cases of severe burns. Therefore, the present study aimed to provide valuable information for a better control of infections in the event of such disasters. In this study, a total of 16 patients who suffered extremely severe burns after an LPG tanker explosion were included. Thereafter, bacteriological culture results were collected within a week. Of 16 patients, 13 (81.25%) were male and the average age of all patients was 60.63 years. In addition, the mean burned area was 83.03% TBSA. Additionally, a total of 553 organism cultures were conducted out of which 287 isolates (51.90%) showed positive results. Notably, 38.52% were Gram-negative bacteria, 7.59% were Gram-positive bacteria and 5.79% were fungi. Moreover, the most prevalent Gram-negative bacteria were *Stenotrophomonas maltophilia* (28.97%) followed by *Acinetobacter baumannii* (28.53%), and *Klebsiella pneumoniae* (14.02%). On the other hand, the three most predominant Gram-positive bacteria were *Enterococcus faecalis* (33.33%), *Staphylococcus aureus* (28.89%) and *Staphylococcus sciuri* (17.78%). Furthermore, the most common fungi included *Candida* (38.24%), *Fusarium* (20.59%) and *Aspergillus fumigatus* (14.71%). With regard to the bacterial resistance patterns, carbapenem-resistant organisms included *Acinetobacter baumannii* (97.80%), *Pseudomonas aeruginosa* (67.57%), and *Klebsiella pneumoniae* (75.56%). In addition, *Staphylococcus sciuri*, *Staphylococcus epidermidis*, and *Staphylococcus haemolyticus* were identified to be methicillin-resistant. This study revealed that there was a high incidence of infection in victims of severe burns as a result of mass burn accidents, accompanied by early fungal infection.

## Introduction

Liquefied petroleum gas (LPG)-related burns are on the increase over the recent years and therefore pose a significant threat to numerous individuals [1, 2]. Notably, several such disasters have been reported in both Turkey [3, 4] and India [5] all of which left dozens of injured victims. However, information with regard to LPG tanker explosions in China remains largely scarce. Nonetheless, an unfortunate LPG tanker explosion occurred on the highway in eastern China in June 2020 and injured nearly 200 people including nearby local residents. The explosion was so profound that a few victims were found in distant rice fields away from the site where it occurred.

Despite improvements in fluid resuscitation, wound management and nutritional support, the survival rate of patients with severe burns is still low. In addition, given the lack of a skin barrier and immunosuppression, burn patients are more vulnerable to bacterial infections. These infections may in turn lead to a high cost of treatment and prolonged periods of hospitalization [6]. Moreover, it should be noted that infections are currently among the most dangerous complications in burn patients, especially those with extremely severe burns [6–8]. Therefore, proactive and empirical control of infections is critical for the management of burn patients. However, little information exists regarding the early microbiological patterns in severe burns arising from accidents such LPG tanker explosions. Consequently, the present study sought to describe the epidemiological characteristics and early bacteriological profiles of mass burn patients. This may provide valuable information for the timely and effective control of infection in similar disasters.

## Patients and Methods

17 burn patients in poor condition were transferred to Second Affiliated Hospital of Zhejiang University, College of Medicine for further treatment, having stayed in their local hospital for 1–2 days. The patients received specialized treatment from a multidisciplinary team including burn surgeons, infectious diseases physicians and intensive care unit specialists. Based on the Chinese severity of burns standards, an extremely severe burn was defined as a burn area > 50% Total Body Surface Area (TBSA) or > 20% TBSA in a third-degree burn [1]. Consequently, 16 patients with a burn area of > 50% TBSA were included in this study and patient data were collected from the database. The variables included epidemiological data (sex, age), injury features (%TBSA, sites of burns, extent of burns, inhalation injury and burns score), treatment characteristics (tracheotomy, hours prior to admission and time to first excision) and bacteriological culture results within a week (isolated bacteria and bacterial resistance patterns). Additionally, all continuous variables were expressed as means and interquartile ranges while categorical variables were reported as numbers and percentages.

## Results

### Demographics

The average age of the patients was 60.63 (IQR 57.75–76) years out of which 11 (68.75%) were older than 60 years (Table 1) and 13 (81.25%) were male. In addition, the mean burnt area was 83.03% TBSA (IQR 80–95%) and the burn percentage of 11 patients (68.75%) was more than 90% TBSA. Moreover, the full-thickness burns in 14 patients (87.5%) were more than 50% TBSA, with a median full-thickness burns of 71.97% TBSA (IQR 59.5–92). Notably, except for the hips and perineum the rest of the sites were burned in all the patients. Additionally, the ABSI, revised Baux score and PBI were 13.06, 164.59 and 142.55, respectively. Furthermore, almost all the patients (93.75%) had inhalation injury and received tracheotomy where the time of first operation was 3.63 days (IQR 3–4). Finally, all the patients received escharotomy and heterologous skin grafting as a temporary measure. Of these 16 patients, 7 patients died, the overall mortality rate was 43.75%.

**Table 1:** Demographic, injury and treatment characteristics of extremely severe burns

	Cases	
	Number	Percentage (%)
Sex		
Male	13	81.25
Female	3	18.75
Age (years)		
< 60	5	31.25
≥ 60, < 80	9	56.25
≥ 80	2	12.5
Median (years)	60.63 (57.75, 76)	
TBSA (%)		
< 80	4	25
≥ 80, < 90	1	6.25
≥ 90	11	68.75
Median (%)	83.03 (80,95)	
Full thickness burns		
≥ 30, < 50	2	12.5
≥ 50, < 80	7	43.75
≥ 80	7	43.75
Median (%)	71.97 (59.5, 92)	
Sites of burns		
Head/face/neck	16	19.28
Upper limbs	16	19.28
Trunk	16	19.28
Lower limbs	16	19.28
Hips	12	14.46
Perineum	7	8.43
Inhalation injury	15	93.75
Tracheotomy	15	93.75
Burn scores		
ABSI	13.06 (12.75, 15)	
Baux score	164.59 (150.13, 185.25)	
PBI	142.55 (131.38, 165.75)	
Hours prior to admission, hours	2.4 (1.61, 2.58)^a^	
Time to first excision (days)	3.63 (3, 4)	
Treatment outcome		
Survived	9	56.25
Died	7	43.75

Data are median and interquartile range (IQR) or number and percentage

^a^Data of two patients were missing

### Bacteriology

A total of 553 organism cultures were conducted and positive results were obtained from 287 (51.90%) isolates while 266 (48.10%) were negative. Of 287 positive isolates, 38.52% were Gram-negative bacteria, 7.59% Gram-positive bacteria and 5.79% fungi (Table 2). In addition, the median duration of detecting the first Gram-positive bacteria, Gram-negative bacteria and fungi was 1.86, 2.27 and 1.75 days, respectively.

**Table 2:** Positive culture distribution of extremely severe burns

Organisms	Wound swab	Sputum	Blood	CVC	Urine	Feces	Total
	201	144	73	54	46	35	553
Gram-negative	91 (45%)	104 (72%)	6 (8%)	1 (2%)	0 (0%)	11 (31%)	213 (39%)
Gram-positive	33 (16%)	3 (2%)	5 (7%)	0 (0%)	0 (0%)	1 (3%)	42 (8%)
Fungi	16 (8%)	9 (6%)	0 (0%)	0 (0%)	6 (13%)	1 (3%)	32 (6%)
Total	140 (70%)	116 (81%)	11 (15%)	1 (2%)	6 (13%)	13 (37%)	287 (52%)

*CVC* central venous catheter

The most prevalent Gram-negative bacteria were *Stenotrophomonas maltophilia* (28.97%) followed by *Acinetobacter baumannii* (28.53%), *Klebsiella pneumoniae* (14.02%) and *Pseudomonas aeruginosa* (11.53%). Additionally, *Acinetobacter baumannii* was the most prevalent organism cultured from wounds (29.55%) and blood (50%) while *Stenotrophomonas maltophilia* was the most predominant isolate from sputum (32.34%). On the other hand, the most common pathogen from feces was *Klebsiella pneumoniae* (40%) although no organism was cultured from urine (Table 3). With regard to the bacterial resistance pattern, the carbapenem-resistant organisms included *Acinetobacter baumannii* (97.80%), *Pseudomonas aeruginosa* (67.57%), *Klebsiella pneumoniae* (75.56%), *Serratia marcescens* (100%) and *Escherichia coli* (66.67%). Moreover, *Escherichia coli* showed resistance to almost all the tested families of antibiotics (Table 4). *Acinetobacter baumannii* also showed high resistance to all the studied families of antibiotics, except for Tigecycline (5.62%), Amikacin (5.62%) and Minocycline (13.48%). Notably, apart from Ciprofloxacin (20%), *Pseudomonas Aeruginosa* also showed high resistance. Furthermore, *Klebsiella Pneumoniae* was shown to be multidrug-resistant, except in Ticarcillin–clavulanic acid (8.82%), Amikacin (20.59%), Trimethoprim/sulfamethoxazole (8.82%) and Ciprofloxacin (8.82%). Interestingly, most of *Acinetobacter baumannii* and *Pseudomonas aeruginosa* was sensitive to colistin. Colistin sensitivity of *Acinetobacter Baumannii* and *Pseudomonas Aeruginosa* was 70% and 84%, respectively. Of these 16 patients, almost all patients (15 patients, 93.75%) were treated with colistin. The survival rate of patients treated with colistin was 53.33% (8 patients), and 11 patients (73.33%) required renal replacement therapy in these patients.

**Table 3:** Bacterial distribution from different positive cultures

Organisms	Wound swab	Sputum	Blood	CVC	Urine	Feces		Total
Gram-negative							Carbapenem-resistant	
*Acinetobacter baumannii*	39 (30%)	45 (27%)	3 (50%)	0 (0%)	0 (0%)	4 (27%)	89 (98%)	91 (29%)
*Klebsiella pneumoniae*	14 (11%)	25 (15%)	0 (0%)	0 (0%)	0 (0%)	6 (40%)	34 (76%)	45 (14%)
*Pseudomonasaeruginosa*	12 (9%)	25 (15%)	0 (0%)	0 (0%)	0 (0%)	0 (0%)	25 (68%)	37 (12%)
*Stenotrophomonas maltophilia*	38 (29%)	54 (32%)	1 (17%)	0 (0%)	0 (0%)	0 (0%)	0 (0%)	93 (29%)
*Serratia marcescens*	0 (0%)	7 (4%)	0 (0%)	0 (0%)	0 (0%)	0 (0%)	7 (100%)	7 (2%)
*Escherichia coli*	1 (1%)	0 (0%)	0 (0%)	0 (0%)	0 (0%)	2 (13%)	2 (67%)	3 (1%)
Others^a^	28 (21%)	11 (7%)	2 (33%)	1 (100%)	0 (0%)	3 (20%)	0 (0%)	45 (14%)
Total	132	167	6	1	0	15	158 (49%)	321
Gram-positive							Methicillin-resistant	
*Enterococcus faecalis*	11 (31%)	2 (67%)	2 (40%)	0 (0%)	0 (0%)	0 (0%)	0 (0%)	15 (33%)
*Staphylococcus aureus*	10 (28%)	1 (33%)	1 (20%)	0 (0%)	0 (0%)	1 (100%)	7 (54%)	13 (29%)
*Staphylococcus epidermidis*	3 (8%)	0 (0%)	0 (0%)	0 (0%)	0 (0%)	0 (0%)	3 (100%)	3 (7%)
*Staphylococcus haemolyticus*	2 (6%)	0 (0%)	0 (0%)	0 (0%)	0 (0%)	0 (0%)	2 (100%)	2 (4%)
*Staphylococcus sciuri*	6 (17%)	0 (0%)	2 (40%)	0 (0%)	0 (0%)	0 (0%)	8 (100%)	8 (18%)
Others^b^	4 (11%)	0 (0%)	0 (0%)	0 (0%)	0 (0%)	0 (0%)	0 (0%)	4 (9%)
Total	36	3	5	0	0	1	20 (44%)	45
Fungi								
*Candida*^c^	1 (6%)	5 (56%)	0 (0%)	0 (0%)	6 (100%)	1 (100%)		13 (38%)
*Aspergillus fumigatus*	1 (6%)	4 (44%)	0 (0%)	0 (0%)	0 (0%)	0 (0%)		5 (15%)
*Fusarium*	7 (39%)	0 (0%)	0 (0%)	0 (0%)	0 (0%)	0 (0%)		7 (21%)
Others^d^	9 (50%)	0 (0%)	0 (0%)	0 (0%)	0 (0%)	0 (0%)		9 (27%)
Total	18	9	0	0	6	1		34

^a^Others included *Aeromonas hydrophila*, *Enterobacter cloacae*, *Flavobacterium odoratum*, *Citrobacter freundii*, *Pseudomonas putida*, *Proteus mirabilis*, *Enterobacter aerogenes*, *Proteus penneri*, *Klebsiella oxytoca*, *Ralstonia mannitolilytica *and* Burkholderia cepacian*

^b^Others included *Bacillus subtilis*, *Staphylococcus lugdunensis *and* Enterococcus gallinarum*

^c^Candida included *Candida tropical*, *Candida albicans*, *Candida parapsilosis *and* Candida famata*

^d^Others included *Cunninghamella bertholletiae*, *Alternaria Nees*, *Rhizopus*, *Mucor *and* Flavomyces*

**Table 4:** Bacterial resistance pattern and colistin sensitivity of carbapenem-resistant organisms

Antimicrobial resistance	*Acinetobacter baumannii* (*n* = 91)	*Pseudomonas aeruginosa* (*n* = 37)	*Klebsiella pneumoniae* (*n* = 45)	*Serratia marcescens* (*n* = 7)	*Escherichia coli* (*n* = 3)
Carbapenem-resistant	89 (98%)	25 (68%)	34 (76%)	7 (100%)	2 (67%)
Tigecycline	5 (6%)	25 (100%)	–	–	–
Imipenem	87 (98%)	25 (100%)	34 (100%)	–	2 (100%)
Meropenem	56 (63%)	24 (96%)	–	4 (57%)	–
Ertapenem	–	–	31 (91%)	3 (43%)	2 (100%)
Piperacillin–tazobactam	89 (100%)	24 (96%)	34 (100%)	–	2 (100%)
Cefperazone–sulbactam	69 (78%)	24 (96%)	31 (91%)	7 (100%)	2 (100%)
Amoxicillin–clavulanic acid	–	–	31 (91%)	3 (43%)	2 (100%)
Ticarcillin–clavulanic acid	56 (63%)	24 (96%)	3 (89%)	4 (57%)	–
Aztreonam	–	–	–	4 (57%)	–
Cefuroxime Sodium	–	–	31 (91%)	3 (43%)	–
Cefuroxime axetil	–	–	31 (91%)	3 (43%)	2 (100%)
Cefoxitin	–	–	31 (91%)	3 (43%)	2 (100%)
Cefatriaxone	33 (37%)	–	31 (91%)	3 (43%)	2 (100%)
Ceftazidine	89 (100%)	15 (60%)	34 (100%)	4 (57%)	2 (100%)
Cefepime	82 (92%)	15 (60%)	22 (65%)	–	2 (100%)
Tobramycin	37 (42%)	–	–	4 (57%)	–
Amikacin	5 (6%)	–	7 (21%)	–	–
Minocycline	12 (13%)	–	–	–	–
Doxycycline	53 (60%)	–	–	–	–
Trimethoprim/sulfamethoxazole	74 (84%)	–	3 (9%)	3 (43%)	2 (100%)
Levofloxacin	80 (90%)	16 (64%)	34 (100%)	7 (100%)	2 (100%)
Ciprofloxacin	56 (63%)	5 (20%)	3 (9%)	4 (57%)	–
Colistin sensitivity	62 (70%)	31 (84%)	–	–	–

Moreover, the three most predominant Gram-positive bacteria were *Enterococcus faecalis* (33.33%), *Staphylococcus aureus* (28.89%) and *Staphylococcus sciuri* (17.78%). Notably, *Enterococcus faecalis* was the most common organism isolated from wounds (30.56%), sputum (66.67%) and blood (40%) although no Gram-positive organism was detected in the Central Venous Catheter (CVC) and urine (Table 3). In addition, all the *staphylococcus sciuri* isolates and 53.85% of *staphylococcus aureus* were methicillin-resistant. Other methicillin-resistant organisms in the study included *staphylococcus epidermidis* (100%) and *staphylococcus haemolyticus* (100%). Moreover, *Staphylococcus* showed relatively high resistance to penicillin antibiotics (95%), quinolones, erythromycin (95%), Trimethoprim/sulfamethoxazole (63.64%) and rifampin (50%) as shown in Table 5. However, no resistance to vancomycin and tigecycline was observed.

**Table 5:** Bacterial resistance pattern of methicillin-resistant organisms

Antimicrobial resistance	*Staphylococcus sciuri* (*n* = 8)	*Staphylococcus epidermidis* (*n* = 3)	*Staphylococcus aureus* (*n* = 13)	*Staphylococcus haemolyticus* (*n* = 2)	Total (*n* = 26)
Methicillin-resistant	8 (100%)	3 (100%)	7 (54%)	2 (100%)	20 (77%)
Penicillin	8 (100%)	3 (100%)	7 (100%)	1 (50%)	19 (95%)
Oxacillin	8 (100%)	3 (100%)	7 (100%)	2 (100%)	20 (100%)
Gentamicin	–	2 (67%)	–	–	2 (67%)
Moxifloxacin	4 (50%)	1 (33%)	7 (100%)	–	12 (50%)
Ciprofloxacin	4 (50%)	1 (33%)	7 (100%)	1 (50%)	13 (65%)
Levofloxacin	4 (50%)	3 (100%)	7 (100%)	1 (50%)	15 (75%)
Erythromycin	7 (88%)	3 (100%)	7 (100%)	2 (100%)	19 (95%)
Clindamycin	5 (63%)	3 (100%)	7 (100%)	2 (100%)	17 (85%)
Tetracycline	4 (50%)	1 (33%)	–	1 (50%)	6 (46%)
Linezolid	–	–	–	1 (50%)	1 (50%)
Quinupristin–dalfopristin	–	–	–	1 (50%)	1 (50%)
Trimethoprim/sulfamethoxazole	4 (50%)	3 (100%)	–	–	7 (64%)
Rifampin	–	–	–	1 (50%)	1 (50%)

On the other hand, the most common fungi were *candida* (38.24%), *Fusarium* (20.59%) and *Aspergillus fumigatus* (14.71%). Most of the fungi were also recovered from wounds where *Fusarium* (38.89%) was the most prevalent isolate (Table 3).

## Discussion

A mass burn accident is defined as more than 10 victims burned from a single incident [9]. LPG is widely used as fuel in many areas. Hence, if a huge amount is released from a tanker, it may be destructive to both the container and its surroundings [3]. Moreover, in the event of a disaster, the effect spreads farther than the local medical capacity and some victims may have poor prognosis because of delayed treatment. Although several studies [10, 11] showed that infection did not independently affect mortality, it was still one of the main challenges in victims of severe burns. Therefore, it is crucial to effectively control infections during the treatment of burn patients.

Previous reports showed that the burn wound was typically colonized with Gram-positive bacteria within 2 days while Gram-negative bacteria colonized the wound after 2–3 days [7, 12], similar to observations from the present study. Additionally, the most isolated organisms were Gram-negative bacteria, corroborating with previous studies [11, 13]. However, *Stenotrophomonas maltophilia* was shown to be one of the most common Gram-negative organisms, contrary to previous findings [7, 14] which reported that *Acinetobacter baumannii* was the predominant Gram-negative bacteria. Moreover, *Enterococcus faecalis* (33.33%) was the predominant Gram-positive bacterium while *Staphylococcus aureus* (28.89%) ranked second. It is noteworthy that the numbers of the two bacterial species were very close and this might have been due to the small sample size used in the study. Nonetheless, a 5-year survey on severe burns in South Korea similarly reported that the *Enterococcus* species replaced *Staphylococcus aureus* as the most common Gram-positive bacteria [15].

Additionally, there was an obvious increase in the carbapenem-resistant and methicillin-resistant organisms, consistent with other studies [13, 16, 17]. Notably, a 10-year retrospective study in Turkey showed a decrease in carbapenem resistance form *Pseudomonas aeruginosa* although that in *Acinetobacter baumannii* increased to 94%, over time [18]. In the present study, *Pseudomonas aeruginosa* (67.57%), *Acinetobacter baumannii* (97.80%), *Escherichia coli* (66.67%), *Serratia marcescens* (100%) and *Klebsiella Pneumoniae* (75.56%) were all highly resistant to carbapenem. In addition, several studies have raised concerns over the threat of carbapenem-resistant *Pseudomonas aeruginosa* and *Acinetobacter baumannii*. Additionally, carbapenem-resistant *Escherichia coli* and *Klebsiella Pneumoniae* are potential major health concerns because of limited treatment options and high mortality rates [19]. Fortunately, most of *Acinetobacter baumannii* and *Pseudomonas aeruginosa* were sensitive to colistin. According to a previous study, a combination of colistin and a glycopeptide was shown to be effective in the treatment of carbapenem-resistant Gram-negative organisms [20]. Despite its potential adverse effects on renal function, colistin continues to play a key role as salvage treatment in burn patients. Mariano et al. [21] reported that colistin therapy can lead to clinical success without a significant association with severe renal impairment.

Moreover, the Gram-positive bacteria, i.e., all the *Staphylococcus sciuri* isolates*, Staphylococcus epidermidis*, *Staphylococcus haemolyticus* and 53.85% of *Staphylococcus aureus* were shown to be methicillin-resistant. Compared to a multicenter survey in China [22], *Staphylococcus epidermidis* had a resistance rate that was higher than 60.69% while that of *Staphylococcus aureus* was less than 74.1%. In addition, the Chinese Antimicrobial Resistance Surveillance of Nosocomial infections [23] reported in 2009 that tigecycline had a sensitivity rate of 100% against *Staphylococcus aureus*,* Enterococcus faecalis* and *Enterococcus faecium*. Furthermore, vancomycin, teicoplanin and tigecycline remained the most effective antibiotics against methicillin-resistant organisms. When infection was sustained by antibiotic-resistant strains, patients would be kept in contact isolation. Generally, several preventive measure would be taken, including (1) patients should be kept in a single room, (2) signs of contact isolation should be placed beside the bed, (3) wearing face masks, water-proof gowns and gloves whenever direct contact with either body fluids or wound exudates happens, do hand hygiene before and after contacting patients, (4) patient's room and all items inside like stethoscope, sphygmomanometer, beside tables, bed, etc. should be disinfected at least three times a day and be dedicated, (5) and final disinfection of ward following the discharge of patient, should be entire, and so on.

Moreover, the study observed that fungal infections appeared earlier (after 1.75 days) than those previously reported [6, 24] to present 2 or 3 weeks after admission. In this study, the most common fungus isolated from wounds was *Fusarium*. As a plant pathogen, *Fusarium* widely exists in natural environments, particularly in soil [25]. However, a study in Vietnam reported that humans could also be infected by the fungus through burn injuries. The study isolated the *Fusarium* and *Rhizopus* fungi from a few patients in rice fields. It is therefore possible that the fungi directly accessed the body through damaged skin. Additionally, the severity of burns made it difficult for the innate immune system to effectively fight against fungal infections. Furthermore, the invasive fungal infection was difficult to treat due to its angio-invasion and absence of a viable treatment strategy. Therefore, it was recommended that early attention should be paid to burn victims and necessary measures should be taken if patients were at a high risk of latent fungal infections. In addition, the routine G and GM tests played an important role in determining the use of anti-fungal drugs [26].

Although the 2016 ISBI Practice Guidelines [27] reported that prophylactic systemic antibiotics could not effectively control infection, they should still be recommended for severe burns because extremely severe burn patients are at a high risk of infection. In addition, high %TBSA, full-thickness burn, older age and presence of inhalation injury were shown to be the most significant risk factors for infection [28]. Moreover, early excision, skin grafting and use of topical antimicrobials were proven to substantially reduce the incidence of infection and sepsis [7, 29, 30]. It is recommended that if the patients’ condition allows, early excision should be carried out as soon as possible. Additionally, invasive medical devices, such as CVC and urinary catheters, should be replaced routinely and cultured.

According to a previous report, respiratory tract infections are among the most common complications in burn patients due to the presence of inhalation injury and prolonged mechanical ventilation [8], consistent with our findings. It is highly likely that the loss of skin barrier integrity was the reason behind the frequent wound infections. Additionally, few organisms were cultured from the urine and CVC in this study although central venous and Foley catheters were frequently used in almost all the severe burn patients. This was probably because urinary tract infections often occur later during hospitalization [8]. Previous studies also reported that bloodstream infections appeared at a later stage, although the present study observed that the infections occurred within the first week. This might have been due to a number of reasons. First, a dysfunction in the immune system might have made the burn patients more susceptible to infection. Alternatively, there was little natural skin left on the extremely severe burn patients, leading to infection. Another explanation is that blood samples might have been contaminated.

Furthermore, the study observed various types of pathogens, some of which are rarely isolated in burn patients. This might have been related to possible contact with water or soil and extreme immunosuppression in patients with extremely severe burns. Although these opportunistic pathogens (such as *Flavobacterium odoratum*, *Rhizopus*, *Enterococcus gallinarum* and *Burkholderia cepacian*) have generally low pathogenicity, they may become clinically important in immunosuppressed patients. To the best of our knowledge, *Cunninghamella bertholletiae*, *Alternaria nees* and *Enterococcus gallinarum* have never been reported in burn patients. Additionally, *Staphylococcus lugdunensis* which is a new emerging organism, is a coagulase-negative *Staphylococcus* species that may lead to serious infections [31]. Presently, these emerging pathogens account for a minority of infections in burn patients. However, they may become a significant clinical threat in future because of the high multidrug resistance as well as mortality rates and ubiquity in the natural environment.

Despite the insightful findings, the study had a few limitations. First, the study only described the bacteriology and early patterns of resistance to antibiotics but did not evaluate the factors affecting infection. Second, it was not elucidated whether bacterial infections contributed to death although only a few reports exist on LPG tanker explosions. Nonetheless, this was the first study to describe the epidemiological and bacteriological characteristics of severe burns in an LPG mass burn accident. In addition, some of the pathogens identified are rarely reported in burn patients. Moreover, the burn patients got injured from the same mass burn accident and received a similar treatment, unlike in other studies. Therefore, the study population had a better homogeneity.

## Conclusion

This study partly revealed that the incidence of infection was very high in severe burn patients from mass burn accidents, accompanied by early fungal infection. Notably, pathogens from soil or water should be considered in burn patients with possible water or soil contact. Consequently, empirical broad-spectrum antibiotics should also be recommended for victims of severe burns from mass burn disasters.

## Data Availability

The data that support the findings of this study are available from the corresponding author upon reasonable request.

## Abbreviations

LPG: Liquefied petroleum gas
TBSA: Total body surface area
CVC: Central venous catheter
